# Supplementary material for: The Resection Rate of Synchronously Detected Liver and Lung Metastasis from Colorectal Cancer Is Low—A National Registry-Based Study
Source: Cancers (Basel). 2023 Feb 23;15(5):1434. doi: 10.3390/cancers15051434 (PMC10000535; doi:10.3390/cancers15051434)
Supplement: Supplementary file 1 [file cancers-15-01434-s001.zip › cancers-2213250-supplementary.pdf]

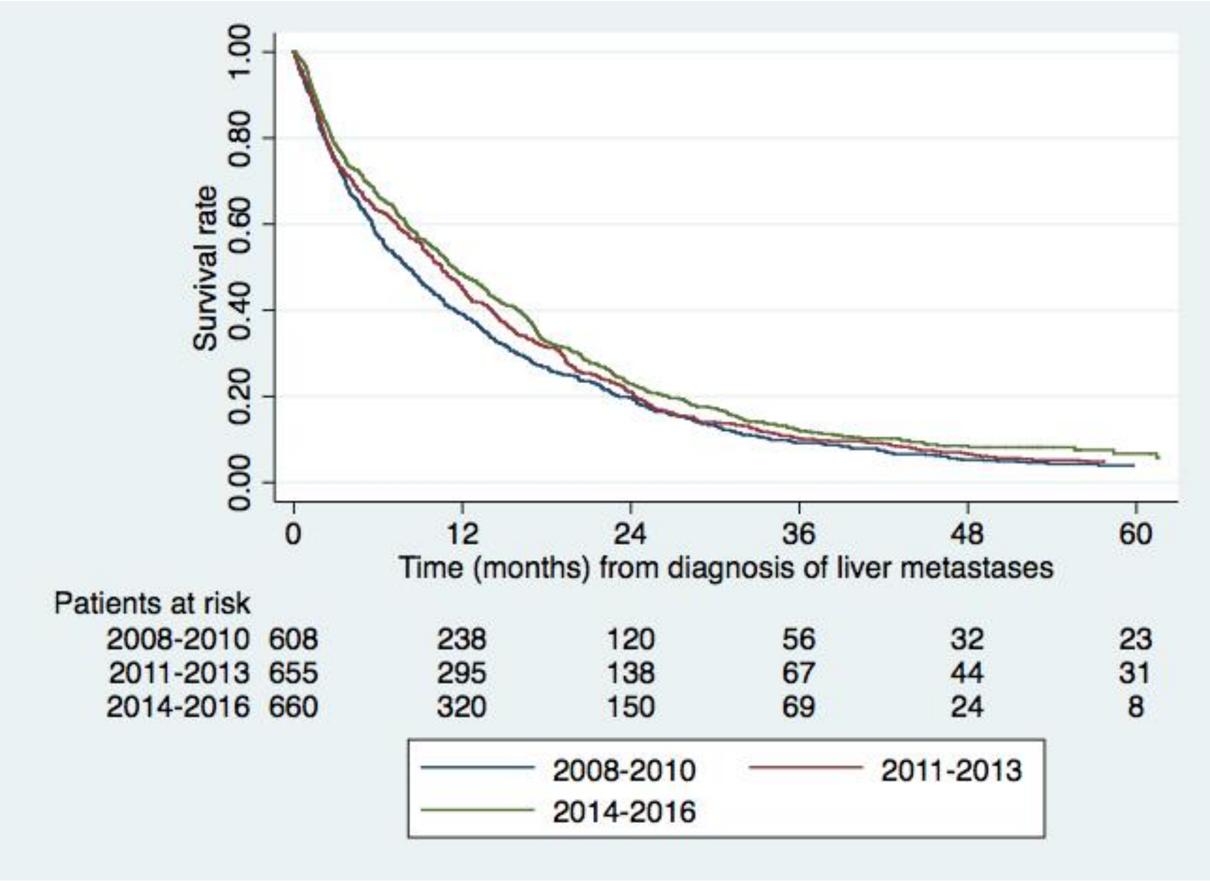

Figure S1. Kaplan-Meier estimates of overall survival irrespective of treatment in different time periods.

Median survival in the entire study cohort was 8 months (95% CI 6.7-9.1 months) in the first time period (2008-2010), 10.5 months (95% CI 9.3-11.7 months) in the second time period (2011-2013) and 11.3 months (95% CI 10.3-13.1) in the third time period (2014-2016), with a significant increase in median survival comparing time period 1 and 3, log-rank test P=0.001.

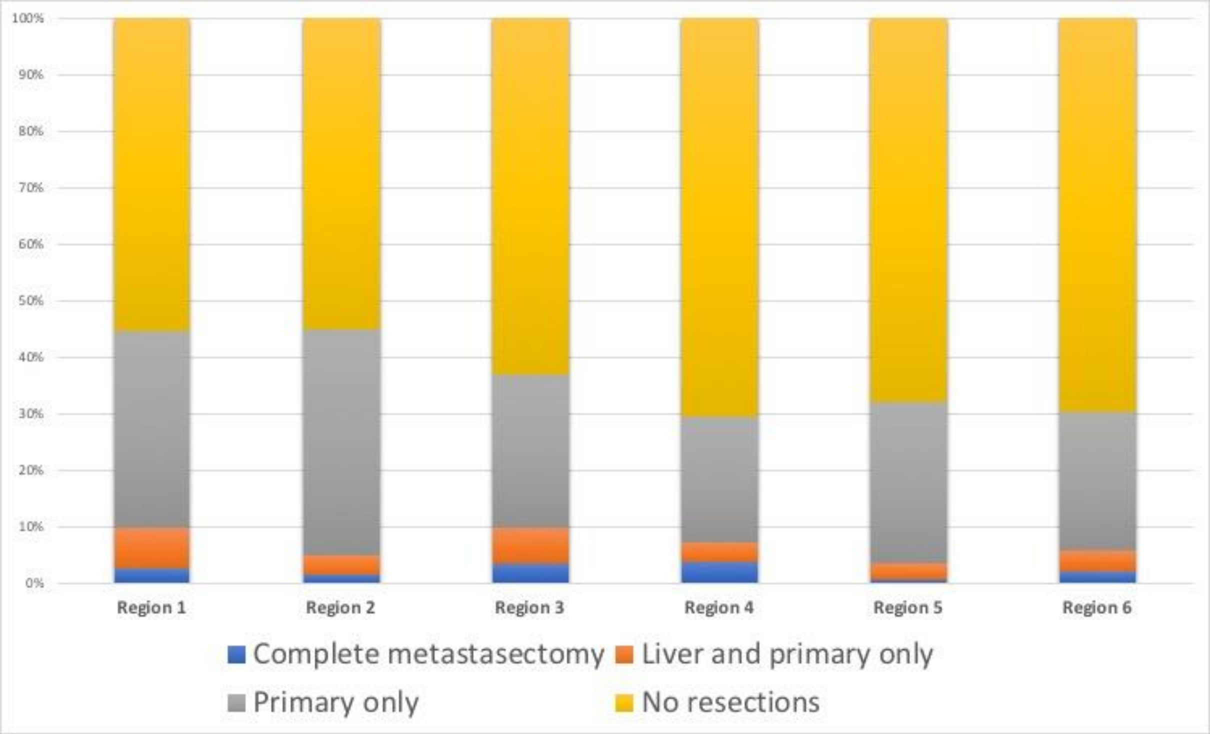

Figure S2. Differences in treatment approach in the six health care regions of Sweden performing liver and lung resection .

Proportional bar chart illustrating the different treatment approaches in the six health care regions of Sweden. The proportion of patient having complete metastasectomy ranged from 0.7% (Region 5) to 3.8% (Region 4), *P*=0. 007
